# A Pair of Multifunctional Cu(II)–Dy(III) Enantiomers with Zero–Field Single–Molecule Magnet Behaviors, Proton Conduction Properties and Magneto–Optical Faraday Effects

**DOI:** 10.3390/molecules28227506

**Published:** 2023-11-09

**Authors:** Shui-Dong Zhu, Yu-Lin Zhou, Fang Liu, Yu Lei, Sui-Jun Liu, He-Rui Wen, Bin Shi, Shi-Yong Zhang, Cai-Ming Liu, Ying-Bing Lu

**Affiliations:** 1 College of Chemistry and Chemical Engineering, Gannan Normal University, Ganzhou 341000, China; zsd2002@sina.com (S.-D.Z.); 19914722345@163.com (F.L.); 18586632742@139.com (Y.L.); zhsy1207@163.com (S.-Y.Z.); 2 Jiangxi Provincial Key Laboratory of Functional Molecular Materials Chemistry, School of Chemistry and Chemical Engineering, Jiangxi University of Science and Technology, Ganzhou 341000, China; 3 Beijing National Laboratory for Molecular Sciences, Key Laboratory of Organic Solids, Chinese Academy of Sciences, Institute of Chemistry, Chinese Academy of Sciences, Beijing 100190, China

**Keywords:** proton conduction, single molecule magnet, magneto–optical faraday effect

## Abstract

Multifunctional materials with a coexistence of proton conduction properties, single–molecule magnet (SMM) behaviors and magneto–optical Faraday effects have rarely been reported. Herein, a new pair of Cu(II)–Dy(III) enantiomers, [DyCu_2_(*RR/SS*–H_2_L)_2_(H_2_O)_4_(NO_3_)_2_]·(NO_3_)·(H_2_O) (***R***–**1** and ***S***–**1**) (H_4_L = [*RR*/*SS*] –N,N′–bis [3–hydroxysalicylidene] –1,2–cyclohexanediamine), has been designed and prepared using homochiral Schiff–base ligands. ***R***–**1** and ***S***–**1** contain linear Cu(II)–Dy(III)–Cu(II) trinuclear units and possess 1D stacking channels within their supramolecular networks. ***R***–**1** and ***S***–**1** display chiral optical activity and strong magneto–optical Faraday effects. Moreover, ***R***–**1** shows a zero–field SMM behavior. In addition, ***R***–**1** demonstrates humidity– and temperature–dependent proton conductivity with optimal values of 1.34 × 10^−4^ S·cm^−1^ under 50 °C and 98% relative humidity (RH), which is related to a 1D extended H–bonded chain constructed by water molecules, nitrate and phenol groups of the *RR*–H_2_L ligand.

## 1. Introduction

Current interest in multifunctional materials with two or more properties in the same matter have been vigorously pursued in chemistry, physics and material science [1,2,3,4] because multifunctionality can simply coexist [5], interact when one function affects the other [6,7] or act in synergy resulting in new functions [8,9], so as to realize efficient, flexible and smart applications. Multifunctional magnets that combine optimized magnetic properties with additional functions are a class of important examples of such interactions or synergies. For instance, chiral magnets are of special interest not only because interactions between chirality and magnetism can cause some intriguing properties, such as multiferroics [10], but also because new physical phenomena could be observed in such optically active magnets stemming from the synergy between chirality and magnetism, such as magneto–chiral dichroisms (MChD) [11,12] and magneto–optical Faraday effects [13]. In addition, proton–conductive magnets have lately gained heightened attention because they could be widely used in electrical and magnetic fields [14,15,16,17], and the coexistence of proton conduction and magnetism is predicted to produce a new property, called spinprotonics [18,19]. In pursuit of advanced multifunctional magnets, tri– or more functional magnets, such as chiral proton–conductive magnets, have also appeared [20]. Nevertheless, such multifunctional magnets with chirality and proton conduction are quite rare because simultaneously integrating these functions into a single material is still a daunting challenge.

Single molecule magnets (SMMs), as one of the major breakthroughs in magnetic research in the past three decades, possess a slow relaxation of magnetization that is similar to memory effects observed in magnetic nanoparticles, offering promising application prospects in the next generation of quantum computing, spintronics and high–density data storage [21,22]. To date, a considerable number of *d*– [23,24,25], *d*–*f* [26,27] and pure *f*–block [28,29,30] metal complexes showing SMM behaviors have been reported. Within this field, lanthanide ions, especially the Dy^3+^ ion, are the most sought–after spin centers because of their ground–state spin and large magnetic anisotropy [31,32]. The magnetic anisotropy of the Dy(III) ion in SMMs arises from spin–orbit coupling and an axial crystal field [33]. For example, high–performance SMMs are Dy(III)–containing metal–inorganic complexes displaying slow magnetic relaxation behavior with record magnetic blocking temperatures [34,35] or high–energy barriers [36]. However, these molecules usually suffer from the fast quantum tunneling of magnetization (QTM) in zero field, limiting the relaxation time [37,38]. Previous achievements demonstrated that intramolecular magnetic exchange in SMMs can powerfully suppress QTM to obtain high working temperatures [39]. Therefore, the construction of mixed 3*d*–4*f* SMMs is desirable because such systems combine the large magnetic anisotropy of lanthanide and the strong magnetic couplings of 3*d* spin centers.

Recently, multifunctional SMMs have been pursued with particular interest because introducing additional properties can provide deep investigations into magnetic behavior [40] to achieve their applications as soon as possible. For instance, luminescent SMMs, with an interplay between magnetic anisotropy and luminescence, are observed on the basis of the electronic structure of 4*f* metal ions [41], and many electrical–conducting [42,43], chiral [44], proton–conductive SMMs [45] have been prepared. In pursuit of advanced multifunctional materials, a few tri– or more functional SMMs have also appeared [46]. Among them, chiral proton–conductive SMMs have attracted much attention as they have promising applications in electrical and magnetic fields and can provide an excellent platform to explore synergies among different functions (e.g., magnetism and chirality, proton conduction and magnetism). Surprisingly, chiral proton–conductive SMMs with magneto–optical Faraday effects are scarce, to the best of our knowledge, only one such related work has been achieved [47].

In addition to spin center, the selection of organic ligands is important to achieve chiral proton–conducting SMMs. In this paper, the ***R***– and ***S***– amine–phenol ligands, (*RR*/*SS*)–N,N′–bis(3–hydroxysalicylidene)–1,2–cyclohexanediamine (H_4_L), are chosen as organic ligands on the basis of the following considerations: (1) according to numerous previously reported works, the Schiff–base ligands are beneficial for the formation of low dimensional 3*d*–4*f* complexes showing SMM behavior [48,49,50]; (2) chiral ligands are prone to induce the structures to crystallize in enantiomeric forms that can lead to new functions, such as MChD and strong magneto–optical Faraday effects in magnetic molecules; and (3) the ligand with four phenol –OH groups is an excellent H–bonding acceptor and donor that favors the formation of rich H–bonding networks in materials, which is constructive for a proton conductor. Herein, a new pair of Cu(II)–Dy(III) enantiomers, [DyCu_2_(*RR/SS*–H_2_L)_2_(H_2_O)_4_(NO_3_)_2_]·(NO_3_)·(H_2_O) (***R***–**1** and ***S***–**1**), is successfully obtained, which contain linear Cu_2_Dy trimers. Due to the enantiomeric structures of ***R***–**1** and ***S***–**1**, the magnetism and proton conductivity of the two isomers are expected to be the same, therefore, only the magnetic and proton–conducting properties of ***R***–**1** were studied. ***R***–**1** shows the zero–field SMM behavior and moderate proton conductivity of 1.34 × 10^−4^ S·cm^−1^ under 50 °C and 98% RH.

## 2. Results and Discussion

### 2.1. X–ray Single–Crystal Structure Determination

Suitable single crystals of ***R***–**1** and ***S***–**1** were used to collect the diffraction data on a Bruker APEX–II CCD and Rigaku CCD diffractometer equipped with graphite–monochromated Mo–Kα radiation (λ = 0.71073 Å) at 293 K, respectively. The raw intensity dates were reduced and collected through the SAINT software 1996 [51]. The olex2 solve structure solution program and the ShelXL–2015 refinement package were applied to solve the two structures [52,53]. The difference Fourier map was used to locate non–hydrogen atoms. Hydrogen atoms were placed in their calculated positions geometrically and refined based on the riding model. No higher space group could be found by the PLATON software from the IUcr website (http://www.iucr.org/ (accessed on 2 November 2023)) [54]. Pertinent crystal data, structure refinement results and selected bond lengths and bond angles of ***R***–**1** and ***S***–**1** were all displayed in Table S1 and Table S2 in the ESI, respectively.

### 2.2. Crystal Structural Descriptions of **R**–***1***

**Figure 1:**
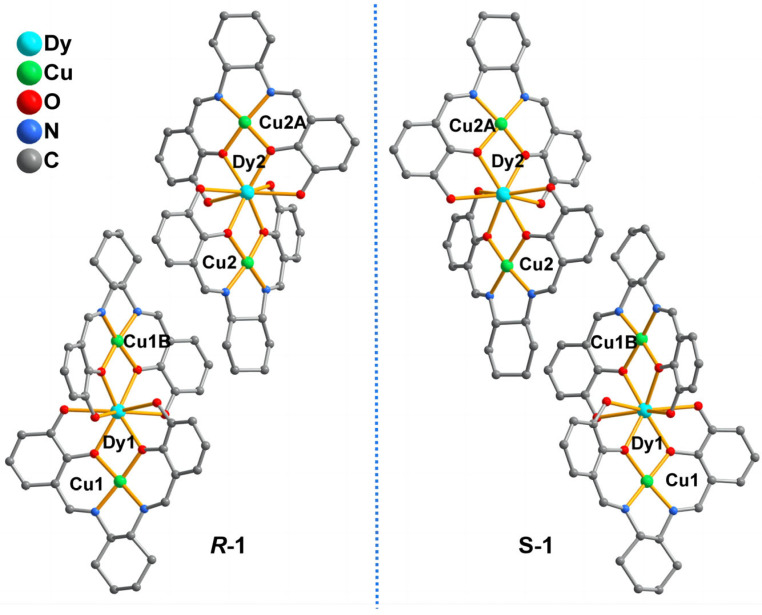
The enantiomeric structures of ***R***–**1** and ***S***–**1**. The coordinated water molecules, coordinated nitrate oxygen atoms and the H atoms are omitted for clarity. Symmetric code: B: 1 − x, y, 2 − z.

Single–crystal X–ray diffraction results show that ***R***–**1** and ***S***–**1** are isomorphous (Figure 1) and crystallized in a monoclinic system with the space group of C2. Therefore, ***R***–**1** is selected as an example to explain the crystal structure. As depicted in Figure S1, the asymmetric unit contains two half–occupied Dy(III) ions, two independent Cu(II) ions, two bideprotonated organic ligands RR–H_2_L^2−^, three NO_3_^−^ anions, four coordinated molecules and one lattice water molecule. The Dy1 ion is coordinated by eight phenolate oxygen atoms (i.e., O1, O2, O3, O4, O1B, O2B, O3B and O4B) from two RR–H_2_L^2−^ ligands and one coordinated water molecule (O1W). Based on the SHAPE analysis [55], the coordinated environment of Dy1 exhibits a capped square antiprism with a continuous shape measurement value of 2.294 (Table S3 and Figure S3a). The Dy2 ion is also nine–coordinated by eight phenolate oxygen atoms and one coordinated water molecule (O5, O6, O7, O8, O5A, O6A, O7A, O8A and O3W) to a similar coordination geometry as the capped square antiprism (Figure S3b). The bond lengths of Dy–O are within the range of 2.311(5)–2.556(7) Å, which are similar to those of Dy–O based complexes [56,57]. Cu1 is pseudo–six coordinated by two phenol O atoms, two nitrogen atoms from one RR–H_2_L^2−^ ligand, an oxygen atom from a nitrate anion and an oxygen atom from a coordinated water molecule, forming a strongly distorted octahedral–coordinated environment (Figure S3c). Notably, one of the “axial” bond lengths (Cu1–O13 = 2.934(4) Å) is substantially longer than the equatorial and another “axial” distances (1.890(5)–1.954(1) Å), which are attributed to the Jahn–Teller distortion and closely resemble other copper(II)–oxygen–based complexes [58,59,60]. Moreover, the distorted octahedron configuration of Cu2 ion is similar to that of the Cu1 ion (Figure S3d). Interestingly, the RR–H_2_L^2–^ adopts a sexadentate ligand with μ_2_–κO1:κO2:κO3:κO4:κN1:κN2 coordination mode, and two phenol groups are deprotonated, resulting in a RR–H_2_L^2–^ deprotonation type. As shown in Figure 1, two RR–H_2_L^2−^ ligands bridge one Dy(III) ion and two Cu(II) ions to form trinuclear Cu1–Dy1–Cu1B and Cu2–Dy2–Cu2B units, in which the Cu(II) ions and the Dy(III) ion are arranged in an almost linear manner with Cu(II)–Dy(II)–Cu(II) angles of 174.046(23)° and 172.975(24)°, respectively.

**Figure 2:**
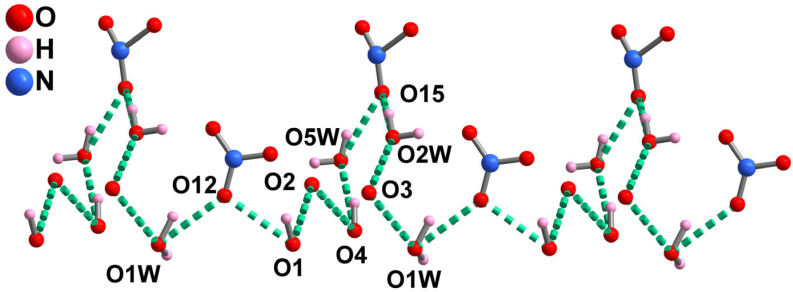
The 1D H–bonding chain and the potential proton transport pathway of ***R***–**1**.

As displayed in Figure S4, these adjacent Cu1–Dy1–Cu1B and Cu2–Dy2–Cu2B trimers are alternately connected by hydrogen bonds constructed with coordinated water molecules, nitrate anions and phenol groups of the RR–H_2_L^2−^ ligand (O1−H1···O12, O1−H1···O13, O1W–H1WA···O12, O1W−H1WB···O12, O2W−H2WA···O14, O4−H4···O5W) forming 1D supermolecular chains. These 1D supermolecular chains are further formed by hydrogen bonds constructed with coordinated water molecules and nitrate anions (O2W−H2WB···O15, O4W−H4WA···O16) forming a 2D supermolecular layer along the bc plane (Figure S5). The NO_3_^−^ counteranions are located in the accessible voids of ***R***–**1** (Figure S5). Notably, a 1D extensive H–bonding chain is formed by water molecules, nitrate anions and phenol groups of the RR–H_2_L^2−^ ligand (O1W−H1WA···O12, O1−H1···O12, O1−H1···O2, O4−H4···O2, O4−H4···O5W, O5W−5WB···O15, O2W−H2WB···O15, O2W−H4···O3, O1W−H4···O3) (Figure 2).

### 2.3. Thermal Stability

As depicted in Figure S6a, the X–ray diffraction patterns of enantiomers ***R***–**1** and ***S***–**1** are basically in accordance with the simulated patterns, demonstrating that the samples are pure phases. TGA curves show that enantiomers ***R***–**1** and ***S***–**1** have similar thermal behaviors. Therefore, the description is performed only for ***R***–**1**, Figure S7 shows that 5.70% of the weight loss before 109 °C is attributed to the release of one lattice water molecule and three coordinated water molecules (calcd. 5.76%). The second weight loss of 14.92% at temperatures up to 297 °C results from the release of three nitrate anions (calcd. 14.88%). Then, the decomposition of the framework occurs until 800 °C. The high thermal stability is key in a promising proton conductor. Thus, the crystal sample of ***R***–**1** is heated to 30 °C, 50 °C, 70 °C, 90 °C and 110 °C in air for 24 h; their measured PXRD patterns still match the simulated one (Figure S6b), indicating that ***R***–**1** has excellent thermal stability.

### 2.4. Circular Dichroism (CD) and Magnetic Circular Dichroism (MCD)

**Figure 3:**
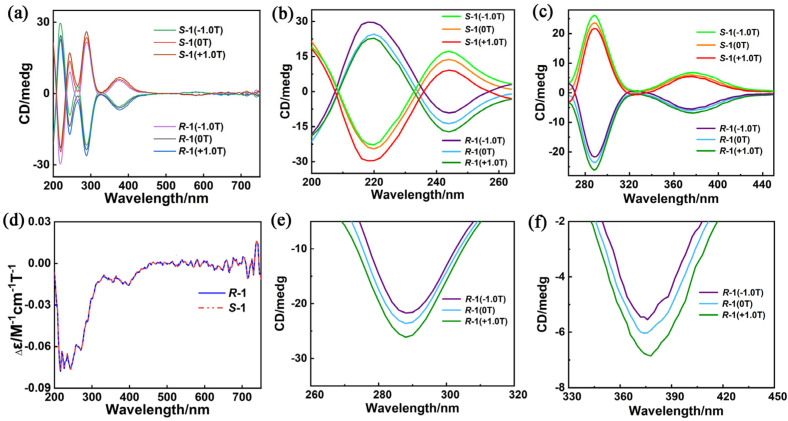
CD spectra of ***R***−**1** and ***S***−**1** in CH_3_CN solution (c = 1.6 × 10*^−^*^5^ molL^−1^; *H* = 0 and ±1.0 T; optical path = 1 mm) in the range of (**a**) 200–750 nm; and partially enlarged view of (**b**) 200–265 nm, (**c**) 265–450 nm and (**d**) MCD spectra of enantiomers ***R***–**1** and ***S***–**1** in a CH_3_CN solution (c = 1.6 × 10*^−^*^5^ molL^−1^) at room temperature; and partially enlarged view of CD spectra of (**e**) ***R***–**1** and (**f**) ***R***–**1** in the range of 260–450 nm.

The UV–vis and CD spectra of ***R***–**1** and ***S***–**1** are obtained to investigate their optical activities. As shown in Figure S9, the UV–vis spectra of ***R***–**1** and ***S***–**1** in CH_3_CN solution are almost identical, and the strong peaks at 274 nm in the UV region can be attributed to the π–π* transition of the aromatic benzene rings of H_4_L ligands. The peak around 352 nm is ascribed to π–π* conjugated interplays between benzene rings and the carbon–nitrogen double bond [61]. The CD spectra of ***R***–**1** and ***S***–**1** in MeCN solutions have an excellent mirror image relationship in the 200–700 nm range, revealing their enantiomeric natures (Figure S8). Moreover, the peak positions of CD spectra are roughly consistent with those in the UV spectra. CD spectra show two cotton peaks around 220 and 244 nm, and a peak around 288 nm, which can be assigned to exciton coupling for π–π* transitions of aromatic groups [62]; additionally, the CD peak at 374 nm is derived from the n–π* transition of azomethine chromophore [63]. The weak peak at 580 nm belongs to the *d*–*d* transition of the transition metal Cu(II) (Figure 3a) [47].

Their CD spectra under external magnetic fields (±1.0 T) and direct current (dc) fields are measured to study the magneto–optical properties of ***R***–**1** and ***S***–**1** at room temperature more clearly. Positive (+1.0 T, NS) and reverse (−1.0 T, SN) magnetic fields are parallel and antiparallel, respectively, to the polarized light. Figure 3a shows that the CD signals of ***R***–**1** and ***S***–**1** exhibit a negative effect under the positive magnetic field (+1.0 T, NS) without changing the position and shape of the peak. By contrast, the negative magnetic field (−1.0 T, SN) shows a positive effect on CD signals. Notably, the CD signal intensities of ***R***–**1** under +1.0 T and ***S***–**1** under −1.0 T are higher than that of CD signals without magnetic field (0 T). As depicted in Figure 3d, pure MCD signals are obtained in accordance with the formula MCD = [CD(+1.0T) − CD(−1.0T)]/2 to further study the magneto–optical properties of ***R***–**1** and ***S***–**1** [64]. Strong MCD signals of ***R***–**1** and ***S***–**1** are detected at 220, 243 and 382 nm (Figure 3b,e), which are ascribed to the large orbital angular momentum of aromatic π–conjugated systems of the H_4_L ligands and excitonic coupling of chromophores [65]. Remarkably, the |g_max_(MCD)| values of ***R***–**1** and ***S***–**1** at room temperature are 0.435 T^−1^ and 0.433 T^−1^, respectively (Figure S10), which are large values and comparable with those of previously reported molecular complexes [47,66], indicating strong magneto–optical Faraday effects.

### 2.5. Magnetic Properties

**Figure 4:**
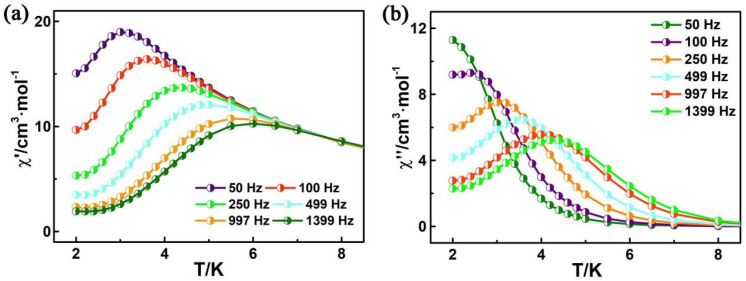
Temperature dependence of the in−phase (**a**) and out−of−phase (**b**) components of the ac magnetic susceptibility for ***R***–**1** in zero–dc fields with an oscillation of 2.5 Oe.

The direct current (dc) magnetic susceptibility has been carried out on polycrystalline samples of ***R***–**1** in the temperature range of 2–300 K, under an applied magnetic field of 1000 Oe (Figure S11a). At room temperature, the χ_M_T molar value is 15.55 cm^3^Kmol^−1^ at 300 K, which is slightly larger than the theoretical value for two Cu^II^ ions (S = 1/2, g = 2, and C = 0.75 cm^3^Kmol^−1^) and one Dy^III^ ion (S = 5/2, L = 5, ^6^H_15/2_, g = 4/3, and C = 14.18 cm^3^Kmol^−1^) [36]. With decreasing temperature, χ_M_T gradually increases within the range of 75–300 K and then quickly rises to a maximum value of 20.78 cm^3^Kmol^−1^ at 6 K. After further cooling, the χ_M_T value of ***R***–**1** decreases to 19.44 cm^3^Kmol^−1^ at 2 K. This behavior may be due to the presence of ferromagnetic Cu–Dy coupling or/and the progressive depopulation of Dy^III^ stark sublevels [67,68]. The field dependence of magnetization (M vs. H) for ***R***–**1** is measured in the temperature range of 2–5 K (Figure S11b). For complex ***R***–**1**, the M value reaches 8.67 Nβ at 2 K, which is lower than the theoretical saturation value of 12 Nβ [69,70]. Furthermore, the unsaturated magnetization and the nonsuperimposition of the M vs. H/T curves suggests the existence of an isolated ground state and/or magnetic anisotropy in the system for compounds [71]. The temperature dependence of the alternating current (ac) magnetic susceptibility measurement was determined to explore the magnetization dynamics of ***R***–**1** in depth. As shown in Figure 4, under a zero–dc field with an oscillation of 2.5 Oe, the plots of in–phase (χ′) and out–of–phase (χ″) signals show temperature dependence in the frequency range of 50–1399 Hz, revealing characteristic magnet relaxation of SMMs [72]. The ln(τ)–T^−1^ plot, which is based on the χ″–ν curves (Figure S12) was fitted using the Arrhenius law, −ln(τ) = −ln(τ_0_) + U_eff_/k_B_T, affording an effective energy barrier of 14.60 K and a pre–exponential factor (τ_0_) of 7.74 × 10^−6^ s for ***R***–**1** (Figure S13a). The energy barrier of ***R***–**1** is comparable with that of the complex ([Dy{hfac}_3_][Cu{hfac}_2_]_2_{3,5–bPy–Ph–Nit}{H_2_O}])_n_·nC_7_H_16_ (hfac^−^ = hexafluoroacetylacetonate, 3,5–bPy–Ph–Nit = 2–[3,5–bis(3–pyridyl)–phenyl]–4,4,5,5–tetramethylimidazoline–1–oxyl–3–oxide, U_eff_ = 17.80 K) [73], and the τ_0_ value of ***R***–**1** is in accordance with the expected τ_0_ values (i.e., 10^−6^–10^−11^) for SMMs [74]. The Cole–Cole diagrams (χ″ vs. χ′) of ***R***–**1** show semicircular shapes between 2.0–4.1 K. The width distribution of relaxation time (α) was fitted with the CC–Fit (Figure S13b), which uses the generalized Debye model, giving α values of 0.14–0.19 (Table S5). The small α values indicate a relatively narrow distribution for a single relaxation process.

### 2.6. Proton Conduction

**Figure 5:**
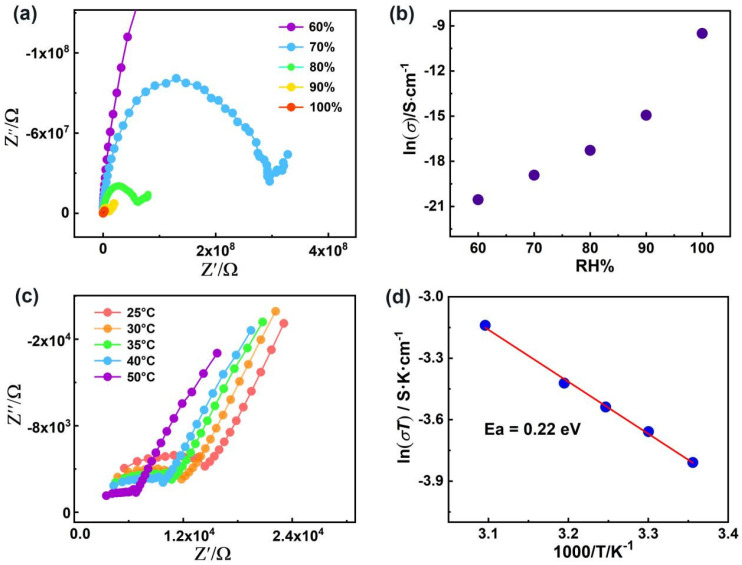
(**a**) Nyquist plot for ***R***−**1** at 25 °C under different RH levels; (**b**) plot of proton conductivity for ***R***−**1** vs. RH at 25 °C; (**c**) Nyquist plot for ***R***−**1** at different temperatures under 100% RH; (**d**) Plots of ln(σT) vs. 1000/T for ***R***−**1** under 100% RH.

In recent years, coordination complexes (CPs), as a new class of proton–conducting materials that can be used in fuel cells, smart grids and information processing devices [75,76], have been highlighted because of their low cost, moderate operating temperature, functionalizable pores and tunable structures. In particular, their crystalline features offer excellent opportunities for revealing the structure–activity relationship, such as proton transfer pathways, which is conducive to designing improvements to proton conductive materials [77,78]. Previous reports showed that designable H–bonding networks for proton transport pathways are crucial in a low–temperature proton conductor. Therefore, the 1D extended H–binding chain in ***R***–**1** and ***S***–**1** can act as an excellent proton transport pathway, and the relatively high water absorption and high thermal properties of ***R***–**1** make its good proton conductivity property to be explored. Ac impedance measurement was carried out using a compacted pellet over a frequency domain of 10^7^–1 Hz. As illustrated in Figure 5a, all Nyquist plots present one semicircle at high frequencies with an inclined tail at low frequencies, indicative of a typical feature of proton migration [79,80]. The humidity–dependent proton conductivities of ***R***–**1** are measured under 60%–100% RH at a fixed temperature to evaluate the effect of humidity on conductivity (Figure 5b). At 60% RH and 25 °C, the conductivity of ***R***–**1** is nearly negligible (1.18 × 10^−9^ S·cm^−1^), which increases by nearly four orders of magnitude with increasing RH and evaluated to be 7.44 × 10^−5^ S·cm^−1^ at 100% RH (Table S6). For ***R***–**1**, the increasing trend of proton conductivities manifest that guest water molecules play a remarkable role in promoting the proton migration in accordance with most water–assisted Ln–CPs [81,82]. At fixed RH, the proton conductivity values of ***R***–**1** increase along with increasing temperatures. Temperature–dependent conductivities are explored to further determine the proton conduction mechanism. At 100% RH, the conductivity of ***R***–**1** increases from 7.44 × 10^−5^ S·cm^−1^ to the optimal value of 1.34 × 10^−4^ S·cm^−1^ (under 50 °C) as the temperature increases (Figure 5c and Table S7). The optimal value (1.34 × 10^−4^ S·cm^−1^) is comparable to those of some reported Ln–MOFs, namely, (APP)_4_[BiAgI_8_]·H_2_O (APP = 4–aminopiperidine, 2.09 × 10^−4^ S·cm^−1^ at 95 °C and 90% RH) [83], LnL(H_2_O)_3_·2H_2_O (L = N–phenyl–N′–phenyl bicycle [2,2,2]oct–7–ene–2,3,5,6– tetracarboxdiimide tetracarboxylic acid; 1.33 × 10^−5^, 75 °C, and 97% RH) [84] and Ce(H_5_L)(H_2_O)_4_ (L = 1,2,4,5–tetrakis(phosphonomethyl)benzene, 1.2 × 10^−4^, 85 °C and 95% RH) [85]. As illustrated in Figure 5d, the activation energy (Ea) is calculated to be 0.22 eV by fitting to the Arrhenius equation σT = σ_0_exp(Ea/kT) [86], illustrating that the possible pathway of proton migration for ***R***–**1** follows the Grotthus mechanism (Ea = 0.1–0.4 eV) rather than the vehicle mechanism (Ea = 0.5–0.9 eV). The Grotthus mechanism, a typical hopping mechanism, occurs in the process of proton transfer among hydrogen–bond networks [87,88,89,90]; while the vehicle mechanism is mediated by protons transferred by the diffusion of “movable carriers” (e.g., HS^−^, OH^−^, H_2_O, and H_3_O^+^) [91,92,93]. On the basis of the structural analysis, in ***R***–**1**, the 1–D extended H–bonding chain constructed by water molecules, nitrate anions and phenol groups of the RR–L^2−^ ligand is conducive to high proton conductivity (1.34 × 10^−4^ S·cm^−1^). Remarkably, the optimal proton conductivity of the ***R***–**1** is two orders of magnitude higher than that of the reported chiral proton–conductive SMMs with the magneto–optical Faraday effect (Table S8). Moreover, the well–matched PXRD patterns of ***R***–**1** before and after water conductivity measurements show no change, confirming that the sample is stable during proton conduction measurements (Figure S6a).

## 3. Experimental Section

All chemistry regents were purchased from commercial suppliers and used without further purification. The H_4_L ligand (Figure S2) was synthesized according to the literature [94].

### 3.1. Materials and Instruments

Elemental analysis (C, H, and N) was measured with an Elementar Vario EL III microanalyzer. IR spectra were recorded in the 400–4000 cm^−1^ region using KBr pellets and a Nicolet Magna 750 FT–IR spectrophotometer. PXRD patterns were measured on a Bruker ADVANCE D8θ −2θ diffractometer equipped with Cu–Kα radiation (λ = 1.54057 Å). TG analysis of ***R***–**1** was performed on a NETZSCH STA2500 thermal instrument with heating rates of 10 °C min^−1^ under N_2_ atmosphere from 25 °C to 800 °C. The circular dichroism (CD) spectra were acquired by using a Chirascan ACD spectrometer. The MCD spectra were measured using a Chirascan ACD spectrometer equipped with a permanent magnet (+1.0 T or −1.0 T). Magnetic susceptibilities were carried out on a Quantum Design MPMS–XL5 (SQUID) magnetometer. Proton conductivity measurements were measured on a Solartron 1260 impedance/gain–phase analyzer with a quasi–four–electrode AC impedance technique to study the proton mobility in conditions of different temperatures and humidities. The microcrystalline sample was pressed into a pellet with thickness of 0.71 mm and diameter of 2.5 mm. The resistance values of the sample were obtained from the Debye semicircle on the Nyquist plot. The conductivity was calculated using the equation σ = L/(RS), σ (S·cm^−1^) means the conductivity, L (cm) is the thickness, R (Ω) is the resistance and S (cm^2^) is cross–sectional the area.

### 3.2. Syntheses of **R**–***1*** and **S**–***1***

A mixture of RR–H_4_L (0.2 mmol) and Cu(Ac)_2_·6H_2_O (0.2 mmol) in CH_3_OH/CHCl_3_ (*v*/*v* = 1:1) was stirred for 15 min. Then Dy(NO_3_)_3_·6H_2_O (0.1 mmol) was added and the mixture continuously stirred for another 15 min. After filtration, the filtrate was crystallized at room temperature by evaporation without disturbation. After two weeks, dark purple columnar crystals ***R***–**1** were obtained and washed several times with CHCl_3_. The yield was 45–50% (based on Dy). Complex ***S***–**1** was prepared using the same procedure as the synthesis of ***R***–**1** but SS–H_4_L was used instead of RR–H_4_L. Anal. Calcd for ***R***–**1** (C_40_H_44_Cu_2_DyN_7_O_21_, %): C, 38.42; H, 3.55; N, 7.84%; Found (%): C, 38.56; H, 3.57; N, 7.80%. FT–IR peaks (KBr, cm^−1^) for ***R***–**1**: 423w, 523w, 562w, 648w, 743w, 869w, 1022w, 1209s, 1253s, 1304s, 1383vs, 1467s, 1628vs, 2862m, 2939m, 3404m; for ***S***–**1**: 422w, 525w, 568w, 661w, 744m, 872w, 1021w, 1084w, 1209s, 1248s, 1304s, 1383vs, 1467vs, 1622vs, 2864m, 2937m, 3397m.

## 4. Conclusions

In this work, a new pair of enantiomers containing trinuclear DyCu_2_ linear units, [DyCu_2_(RR/SS–H_2_L)_2_(H_2_O)_4_(NO_3_)_2_]·(NO_3_)·(H_2_O) (***R***–**1** and ***S***–**1**) (H_4_L = [RR/SS]–N,N′–bis [3–hydroxysalicylidene]–1,2–cyclohexanediamine), were rationally designed and successfully prepared. ***R***–**1** shows zero–field SMM behavior, strong magneto–optical Faraday effects and moderate proton conductivity (1.34 × 10^−4^ S·cm^−1^ under 50 °C under 100% RH) originating from a 1D H–bonded chain built by water molecules, nitrate and phenol groups of the RR–H_2_L ligand. Furthermore, this study demonstrates that chiral hydroxysalicylic ligands are good ligands for preparing homochiral 3d–4f molecule–based materials with multifunctionalities including SMM behavior, proton conductivity and magneto–optical coupling.

## Data Availability

X–ray crystallographic data file in CIF format, IR spectra, H–bonding length and angle, selected bond distances and angles, Summary of SHAPE analysis or ***R***–**1** and ***S***–**1**. The asymmetric unit, 1D supramolecular chain, 2D supramolecular layer of ***R***–**1**. The structure of the H_4_L ligand. The PXRD patterns, TGA plot, CD spectra, UV spectra, corresponding g_MCD_ values (MCD) of enantiomers ***R***–**1** and ***S***–**1**. The *χ*_M_*T vs T* plot, ln(*τ*) *vs T*^−1^ plot and field–dependent magnetization for ***R***–**1**. Linear combination of two modified Debye model fitting parameters. The proton conductivity of ***R***–**1** under different RHs and temperatures. CCDC number: ***R***–**1** for 2195312 and ***S***–**1** for 2195311.

## Supplementary Materials

The following supporting information can be downloaded at: https://www.mdpi.com/article/10.3390/molecules28227506/s1. Table S1. Crystal data for ***R*–1** and ***S*–1**. Table S2. Selected bond lengths (Å) and angles (^o^) for ***R*–1** and ***S*–1**. Table S3. Summary of SHAPE analysis for ***R*–1**. Table S4. H-bonding length and angle table for ***R*–1**. Figure S1. The asymmetric unit of ***R*–1** with 40% thermal ellipsoids. Symmetry codes: A: x, 1−y, −z. The H atoms are omitted for clarity. Figure S2. The structure of the H_4_L ligand. Figure S3. The coordinated geometry of Dy1, Cu1, and Cu2. Figure S4. The 1-D supramolecular chain of ***R*–1** connected through hydrogen bonds along the *bc* plane (green dashed line). Figure S5. The 2-D supramolecular layer of ***R*–1** connected through hydrogen bonds along the *ab* plane (green dashed line). Figure S6. (a) PXRD patterns of the simulated one, as-synthesized ***R*–1** and ***S*–1** and after proton conduction of ***R*–1**. (b) PXRD patterns of ***R*–1** after heated at different temperature for 24 h. Figure S7. The TGA plot of ***R*–1** and***S*–1**. Figure S8. CD spectra of enantiomers ***R*–1** and***S*–1** in a CH_3_CN solution (*c* = 0.02 g·L^−1^) at room temperature. Figure S9. UV spectra of enantiomers ***R*–1** and***S*–1** in CH_3_CN solution (*c* = 0.02 g·L^−1^) at room temperature. Figure S10. (a) *χ_M_T* vs *T* plots for ***R*–1** at 1000 Oe. (b) Field-dependent magnetization for ***R*–1**. Figure S11. Plot of ln(*τ*) versus *T*^−1^ for ***R*–1**, the red solid line is fitted with the Arrhenius law. Figure S12. The *χ″-ν* curves for ***R*–1**. Figure S13. (a) Plot of ln(*τ*) versus *T*^−1^ for ***R*–1**, the red solid line is fitted with the Arrhenius law. (b) Cole–Cole plots of ***R*–1** under zero dc field (the yellow solid line represents the least-squares fitting by using CC-FIT software). Table S5. Linear combination of two modified Debye model fitting parameters from 2.0 to 4.1 K at *H*_dc_ = 0 Oe. Table S6. The proton conductivity of ***R*–1** at 25 °C under variable relative humidity (RH). Table S7. The proton conductivity of ***R*–1** at 100 % RH under variable temperature (°C). Table S8. Comparison of the properties of proton conduction, single molecule magnet (SMM) and magneto-optical Faraday effect of ***R*–1** with that of the complexes based on chiral Schiff ligands.
