# Supplementary material for: A Pair of Multifunctional Cu(II)–Dy(III) Enantiomers with Zero–Field Single–Molecule Magnet Behaviors, Proton Conduction Properties and Magneto–Optical Faraday Effects
Source: Molecules. 2023 Nov 9;28(22):7506. doi: 10.3390/molecules28227506 (PMC10673516; doi:10.3390/molecules28227506)
Supplement: Supplementary file 1 [file molecules-28-07506-s001.zip › molecules-2682356-supplementary.pdf]

Supporting Information

A pair of multifunctional Cu(II)-Dy(III) enantiomers with zero-field single molecule magnet behaviours, proton conduction properties and magneto-optical Faraday effects

*Shui-Dong Zhu[1], Yu-Lin Zhou[1], Fang Liu[1], Yu Lei[1], Sui-Jun Liu[2], He-Rui Wen[2], Bin Shi[1], Shi-Yong Zhang[1], Cai-Ming Liu[3,] * and Ying-Bing Lu[1,] **

[1]*College of Chemistry and Chemical Engineering, Gannan Normal University, Ganzhou 341000, Jiangxi P. R. China.*

[2]*School of Chemistry and Chemical Engineering, Jiangxi Provincial Key Laboratory of Functional Molecular Materials Chemistry, Jiangxi University of Science and Technology, Ganzhou 341000, Jiangxi Province, People's Republic of China.*

[3]*Beijing National Laboratory for Molecular Sciences, CAS Key Laboratory of Organic Solids, Institute of Chemistry, Chinese Academy of Sciences, Beijing 100190, P. R. China.*

E-mail: ybluhm@163.com, cmliu@iccas.ac.cn, zsd2002@sina.com

Table S1. Crystal data for ***R*-1** and ***S*-1**.

Table S2. Selected bond lengths (Å) and angles (°) for ***R*-1** and ***S*-1**.

Table S3 Summary of SHAPE analysis for ***R*-1**.

Table S4. H-bonding length and angle table for ***R*-1**.

Figure S1. The asymmetric unit of ***R*-1** with 40% thermal ellipsoids. Symmetry codes: A: *x*, 1−*y*, −*z*. The H atoms are omitted for clarity;

Figure S2. The structure of the H_4L ligand;

Figure S3. The coordinated geometry of Dy1, Cu1, and Cu2.

Figure S4. The 1-D supramolecular chain of ***R*-1** connected through hydrogen bonds along the *bc* plane (green dashed line);

Figure S5. The 2-D supramolecular layer of ***R*-1** connected through hydrogen bonds along the *ab* plane (green dashed line);

Figure S6. (a) PXRD patterns of the simulated one, as-synthesized ***R*-1** and ***S*-1** and after proton conduction of ***R*-1**; (b) PXRD patterns of ***R*-1** after heated at different temperature for 24 hours.

Figure S7. The TGA plot of ***R*-1** and ***S*-1**.

Figure S8. CD spectra of enantiomers ***R*-1** and ***S*-1** in a CH_3CN solution (c = 0.02 g·L^{-1}) at room temperature.

Figure S9. UV spectra of enantiomers ***R*-1** and ***S*-1** in CH_3CN solution (c = 0.02 g·L^{-1}) at room temperature;.

Figure S10. (a) χ_MT *vs* T plots for ***R*-1** at 1000 Oe. (b) Field-dependent magnetization for ***R*-1**.

Figure S11. Plot of ln(τ) versus T^{-1} for ***R*-1**, the red solid line is fitted with the Arrhenius law.

Figure S12. The χ''-ν curves for ***R*-1**.

Figure S13. (a) Plot of ln(τ) versus T^{-1} for ***R*-1**, the red solid line is fitted with the Arrhenius law. (b) Cole–Cole plots of ***R*-1** under zero dc field (the yellow solid line represents the least-squares fitting by using CC-FIT software).

Table S5. Linear combination of two modified Debye model fitting parameters from 2.0 to 4.1 K at H_{dc} = 0 Oe.

Table S6. The proton conductivity of ***R*-1** at 25 °C under variable relative humidity (RH).

Table S7. The proton conductivity of ***R*-1** at 100 % RH under variable temperature (°C).

Table S8. Comparison of the properties of proton conduction, single molecule magnet (SMM) and magneto-optical Faraday effect of ***R*-1** with that of the complexes based on chiral Schiff ligands.

Table S1. Crystal data for ***R*-1** and ***S*-1**.

	***R*-1**	***S*-1**
Formula	$C_{40}H_{48}Cu_2DyN_7O_{21}$	$C_{40}H_{48}Cu_2DyN_7O_{21}$
Fw	1252.43	1252.43
Temp (K)	293(2)	293(2)
Crystal system	monoclinic	monoclinic
Space group	$C2$	$C2$
a, Å	29.2637(12)	29.338(3)
b, Å	8.4552(3)	8.4822(4)
c, Å	24.0200(9)	24.123(2)
β, (deg)	126.913(2)	127.002(13)
D_c, g/cm^3	1.751	1.735
V, Å^3	4751.9(3)	4794.2(9)
Z	4	4
μ (mm^{-1})	2.531	2.509
F (000)	2516.0	2516.0
Reflns collected	34860	10828
Independent reflns	10741	8522
R_{int}	0.0370	0.0167
Theta range, °	5.938–54.938	7.092–58.554
Params/restraints/data	654 / 130/ 10741	654 / 168 / 8522
R_1 [$I > 2\sigma(I)$]	0.0415	0.0439
wR$_2$ (all data)	0.0827	0.0944
GOF on F^2	1.063	1.027
ρ_{max}/ρ_{min}, e Å^{-3}	0.84/ –0.95	0.83 / –0.53

$^aR1 = ||F_o|-|F_c||/|F_o|$; $^bwR2 = [w(F_o^2 - F_c^2)^2]/[w(F_o^2)^2]^{1/2}$

Table S2. Selected bond lengths (Å) and angles (°) for ***R*-1** and ***S*-1**.

Complex ***R*-1** (bond)	lengths (Å)	(angle)	angles (°)
Dy(1)-O(1)	2.488(6)	O(1)-Dy(1)-O(1) #1	76.9(5)
Dy(1)-O(2)	2.309(5)	O(1)#1-Dy(1)-O(4)#1	145.0(3)
Dy(1)-O(3)	2.343(1)	O(1)#1-Dy(1)-O(4)	72.2(4)
Dy(1)-O(4)	2.567(6)	O(1)-Dy(1)-O(4)	145.0(3)
Dy(1)-O1W	2.375(9)	O(1)-Dy(1)-O(4) #1	72.2(4)
Dy(2)-O3W	2.371(11)	O1W-Dy(1)-O(4)	70.4(2)
Dy(2)-O(5)	2.556(7)	O1W-Dy(1)-Cu1	92.98(2)
Dy(2)-O(6)	2.319(6)	O(3)-Cu1-O(2)	82.1(2)
Dy(2)-O(8)	2.483(7)	O3W-Dy(2)-O(8)	141.4(2)
Dy(2)-O(7)	2.311(5)	O6#2-Dy(2)-O3W	76.6(2)
Cu(1)-O2W	2.351(4)	O(6)-Dy(2)-O(5)	62.6(2)
Cu(1)-N(1)	1.954(1)	O(6)#2-Dy(2)-O(5)	108.0(2)
Cu(1)-N(2)	1.909(7)	O(7)#2-Dy(2)-O3W	113.01(2)
Cu(1)-O(2)	1.925(6)	O(6)-Dy(2)-O8	126.6(2)
Cu(1)-O(3)	1.890(5)		
Cu(2)-N(3)	1.921(9)		
Cu(2)-O(6)	1.923(5)		
Complex ***S*-1** (bond)	lengths (Å)	(angle)	angles (°)
Dy(1)-O(1)	2.536(10)	O(3)-Dy(1)-O(3) #1	135.3(5)
Dy(1)-O(1)#1	2.536(10)	O(3)-Dy(1)-O(2)#1	125.8(3)
Dy(1)-O(2)	2.352(10)	O(3)#1-Dy(1)-O(2)#1	64.4(3)
Dy(1)-O(2)#1	2.352(10)	O(3)-Dy(1)-O(2)	64.4(3)
Dy(1)-O(3)	2.309(9)	O(3)#1-Dy(1)-O(2)	125.8(3)
Dy(2)-O(3)W	2.398(18)	O(3)W-Dy(2)-O(5)	141.2(2)
Dy(2)-O(5)	2.493(10)	O(5)-Dy(2)-O(8)	146.0(4)
Dy(2)-O(6)	2.334(11)	O(6)-Dy(2)-O5	63.7(3)
Dy(2)-O(8)	2.588(10)	O(6)-Dy(2)-O3W	114.5(3)
Cu(1)-O2W	2.369(11)	O(6)#2-Dy(2)-O(5)	78.2(4)
Cu(1)-N(1)	1.939(11)		
Cu(2)-N(3)	1.923(13)		

Symmetry Codes for ***R*-1**: #1 1–*x*, +*y*, 2–*z* , #2 1–*x*, +*y*, 1–*z* ; For ***S*-1**: #1 2–*x*, +*y*, 2–*z*, #2 1–*x*, +*y*, 1–*z*.

Table S3 Summary of SHAPE analysis for ***R*-1**.

Metal	label	shape	symmetry	Distortion(τ)
Dy1	EP-9	Enneagon	D_{9h}	35.283
	OPY-9	Octagonal pyramid	C_{8v}	21.351
	HBPY-9	Hexagonal bipyramid	D_{7h}	18.194
	JTC-9	Johnson triangular cupola J3	C_{3v}	15.327
	JCCU-9	Capped cube J8	C_{4v}	9.578
	CCU-9	Spherical-relaxed capped cube	C_{4v}	8.625
	JCSAPR-9	Capped square antiprism J10	C_{4v}	3.607
	**CSAPR-9**	**Capped square antiprism**	$\boldsymbol{C_{4v}}$	**2.264**
	JTCTPR-9	Tricapped trigonal prism J51	D_{3h}	5.320
Dy2	EP-9	Enneagon	D_{9h}	35.045
	OPY-9	Octagonal pyramid	C_{8v}	21.692
	HBPY-9	Hexagonal bipyramid	D_{7h}	17.767
	JTC-9	Johnson triangular cupola J3	C_{3v}	15.306
	JCCU-9	Capped cube J8	C_{4v}	9.022
	CCU-9	Spherical-relaxed capped cube	C_{4v}	8.054
	JCSAPR-9	Capped square antiprism J10	C_{4v}	3.745
	**CSAPR-9**	**Capped square antiprism**	$\boldsymbol{C_{4v}}$	**2.370**
	JTCTPR-9	Tricapped trigonal prism J51	D_{3h}	5.547
Cu1	HP-6	Hexagon	D_{6h}	29.181
	PPY-6	Pentagonal pyramid	C_{5v}	26.936
	**OC-6**	**Octahedron**	$\boldsymbol{O_h}$	**3.460**
	TPR-6	Trigonal prism	D_{3h}	16.876
	JPPY-6	Johnson pentagonal pyramid J2	C_{5v}	28.982
Cu2	HP-6	Hexagon	D_{6h}	31.143
	PPY-6	Pentagonal pyramid	C_{5v}	27.421
	**OC-6**	**Octahedron**	$\boldsymbol{O_h}$	**2.831**
	TPR-6	Trigonal prism	D_{3h}	17.132
	JPPY-6	Johnson pentagonal pyramid J2	C_{5v}	30.002

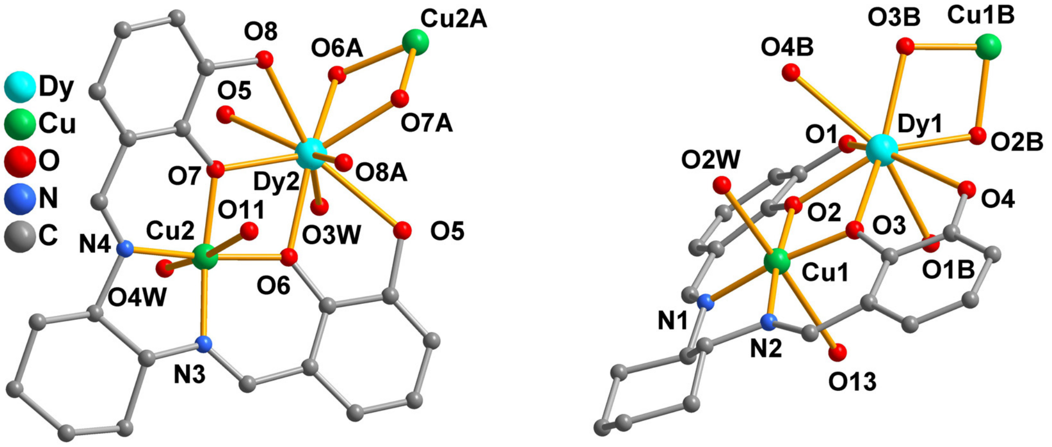

Figure S1. The asymmetric unit of ***R*-1** with 40% thermal ellipsoids. Symmetry codes: A: −*x*, *y*, 1−*z;* B:1−*x*, *y*, 2−*z.* The H atoms are omitted for clarity;

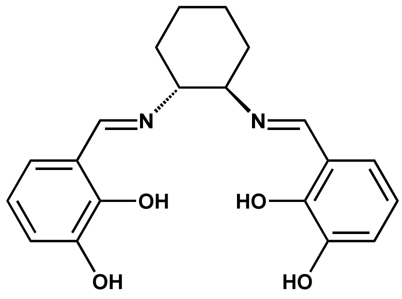

Figure S2. The structure of the H_4L ligand.

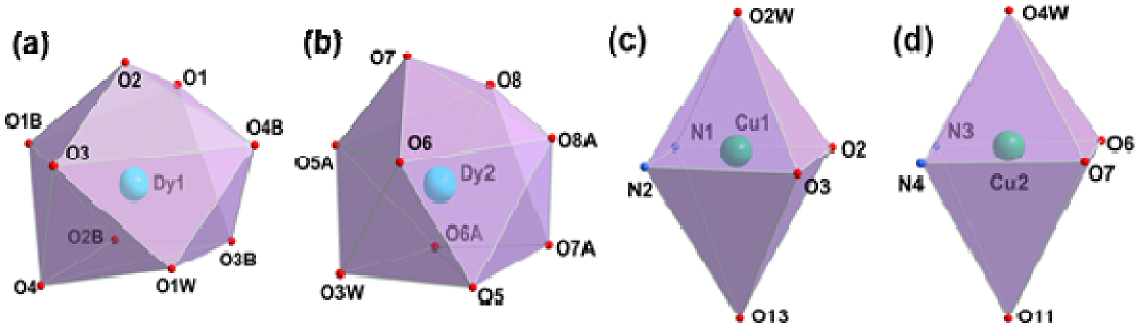

Figure S3. The coordination geometry of Dy1, Dy2, Cu1 and Cu2.

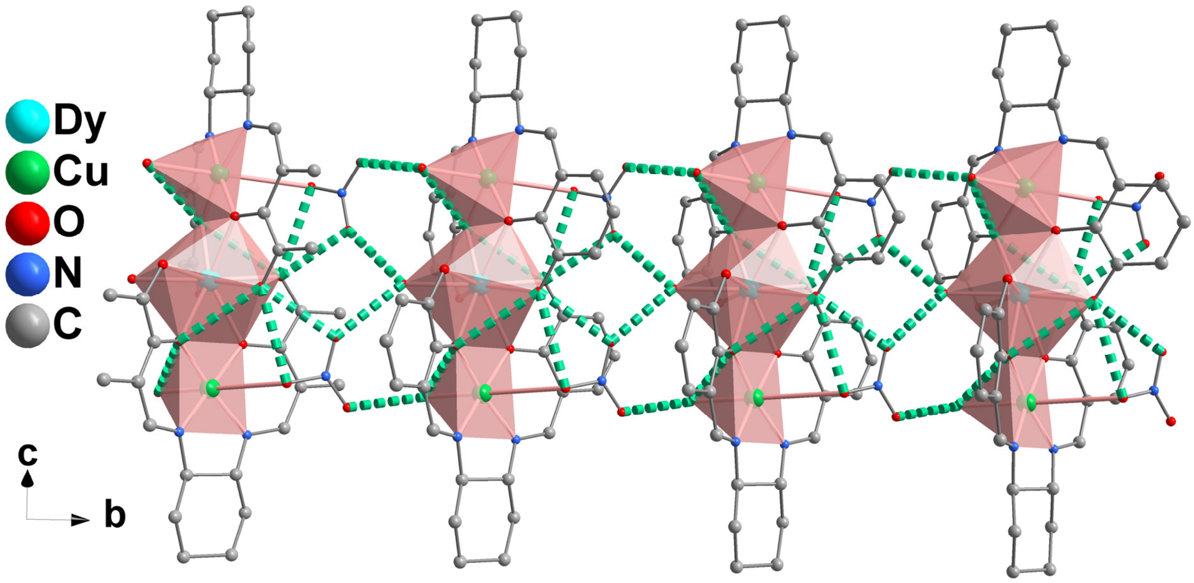

Figure S4. The 1-D supramolecular chain of ***R*-1** connected through hydrogen bonds along the *bc* plane (green dashed line).

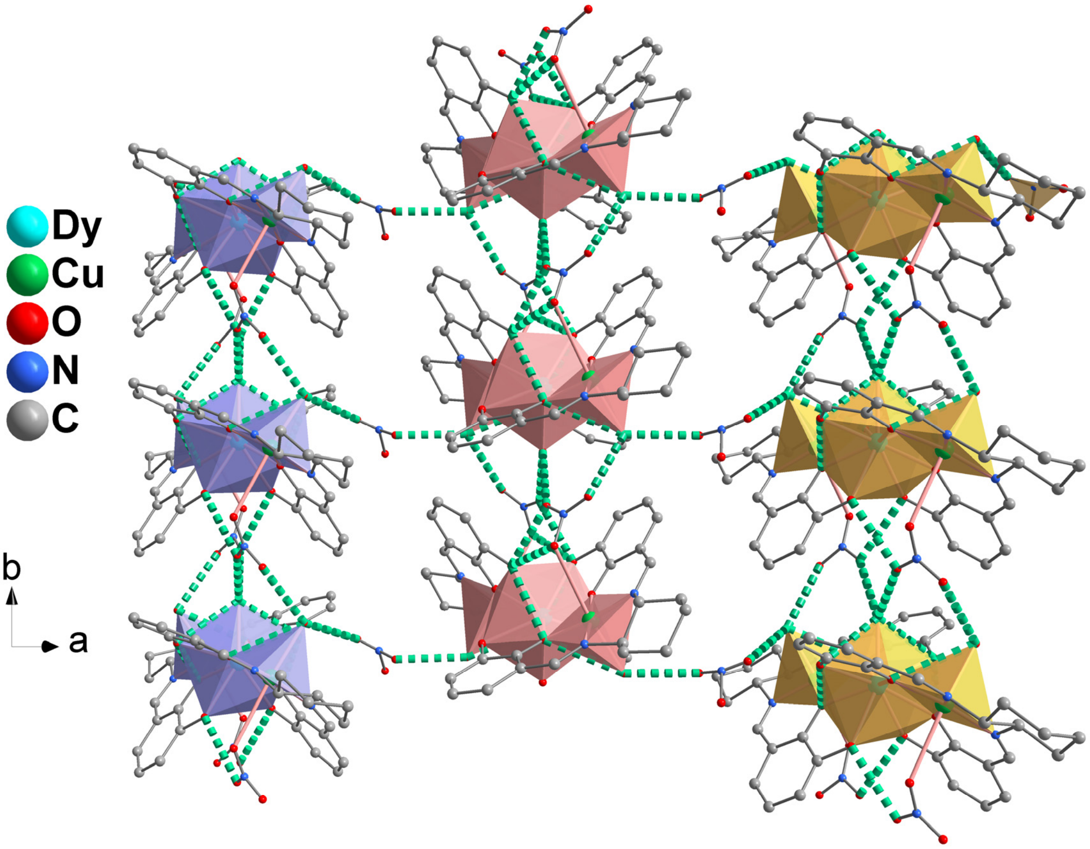

Figure S5. The 2-D supramolecular layer of ***R*-1** connected through hydrogen bonds along the *ab* plane (green dashed line).

Table S4. H-bonding length and angle table for ***R*-1**

D–H···A	d(D…A)(Å)	<DHA(Å)
O1–H1···O12	2.715(18)	118
O1–H1···O13	3.06(2)	161
O1W–H1WA···O12	2.769(18)	132
O1W–H1WB···O12	2.769(18)	133
O2W–H2WA···O14	2.83(3)	168
O4–H4···O5W	2.80(3)	153
O2W–H2WB···O15	2.70(3)	142
O5–H5A···O16	2.56(2)	144(7)
O3W–H3WA···O9	2.76(2)	168
O3W–H3WB···O9	2.76(2)	168
O4W–H4WA···O16	3.19(3)	161
O5W–H5WB···O15	2.68(3)	139
O1–H1···O2	3.0701(1)	84.255(3)
O4–H4···O2	2.8814(1)	84.861(3)
O2W–H4···O3	3.0450(1)	72.206(2)
O1W–H4···O3	2.9086(1)	86.491(2)
O8–H8A···O9	2.87(2)	162(5)
O8–H8A···O11	2.977(18)	137(4)

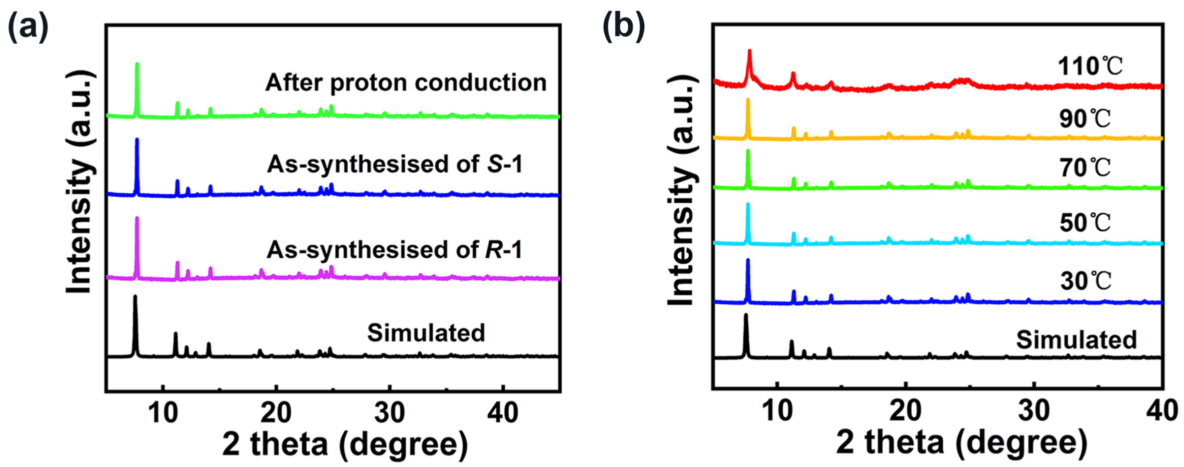

Figure S6. (a) PXRD patterns of the simulated one, as-synthesized ***R*-1** and ***S*-1** and after proton conduction of ***R*-1**; (b) PXRD patterns of ***R*-1** after heated at different temperature for 24 hours.

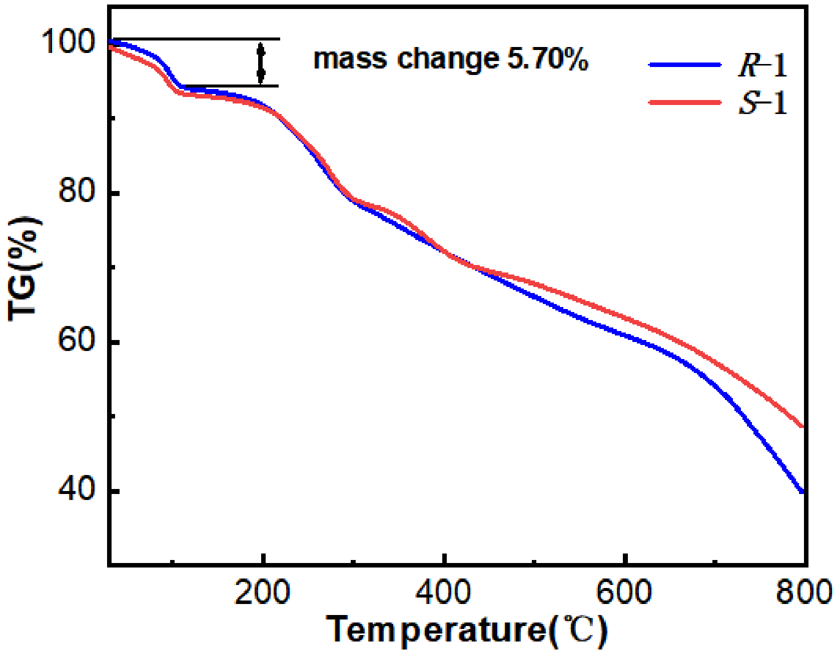

Figure S7. The TGA plot of ***R*-1** and ***S*-1**

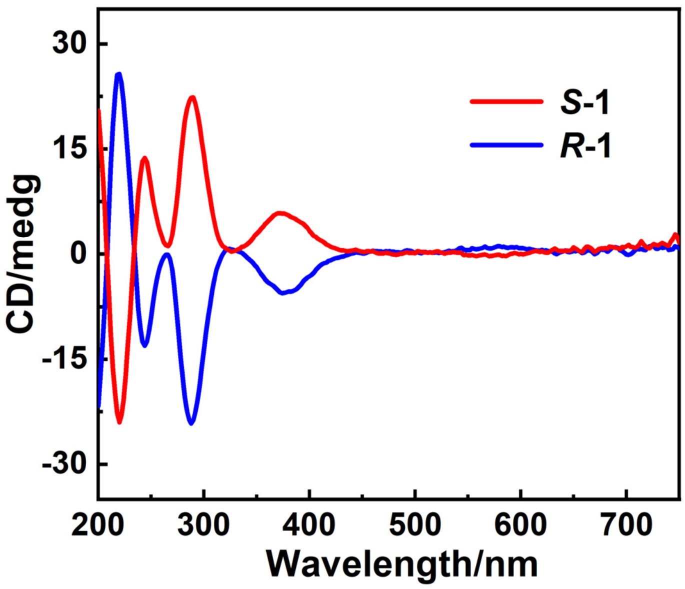

Figure S8. CD spectra of enantiomers ***R*-1** and ***S*-1** in a CH_3CN solution (c = 0.02 g·L^{-1}) at room temperature.

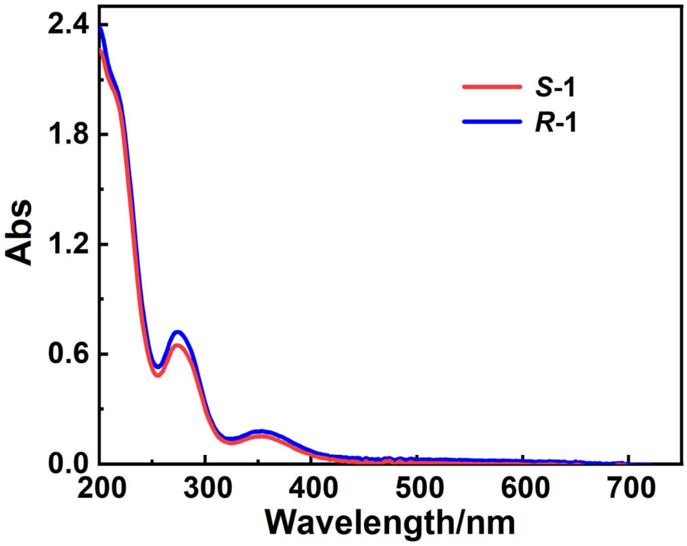

Figure S9. UV spectra of enantiomers ***R*-1** and ***S*-1** in CH_3CN solution (c = 0.02 g·L^{-1}) at room temperature.

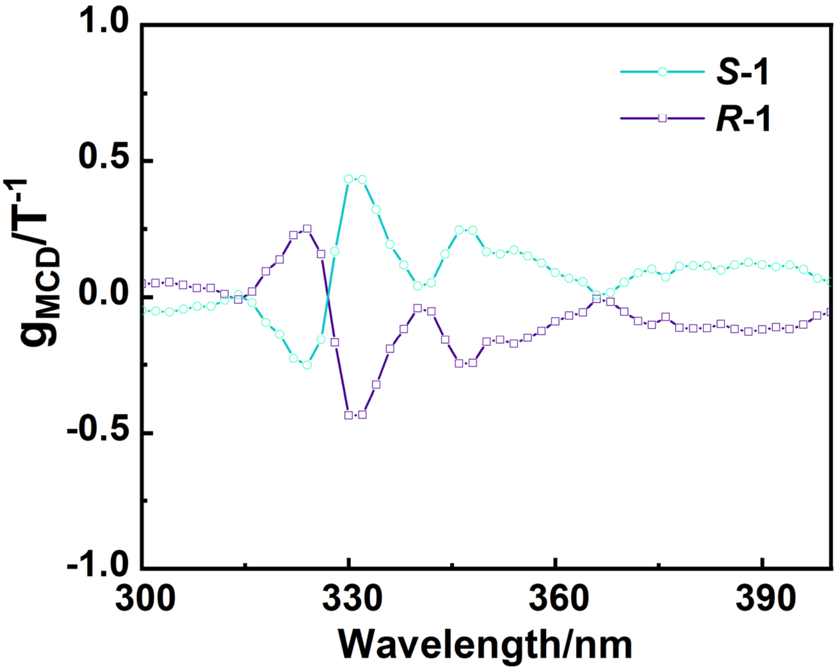

Figure S10. the *g* (MCD) values of ***R*-1** and ***S*-1** at room temperature

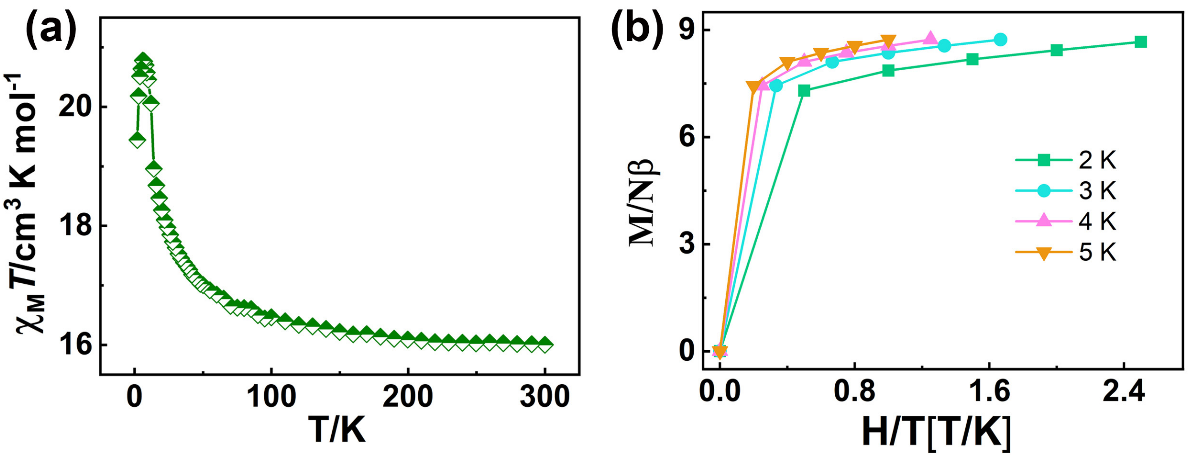

Figure S11. (a) χ_MT *vs* T plots for ***R*-1** at 1000 Oe. (b) Field-dependent magnetization for ***R*-1**.

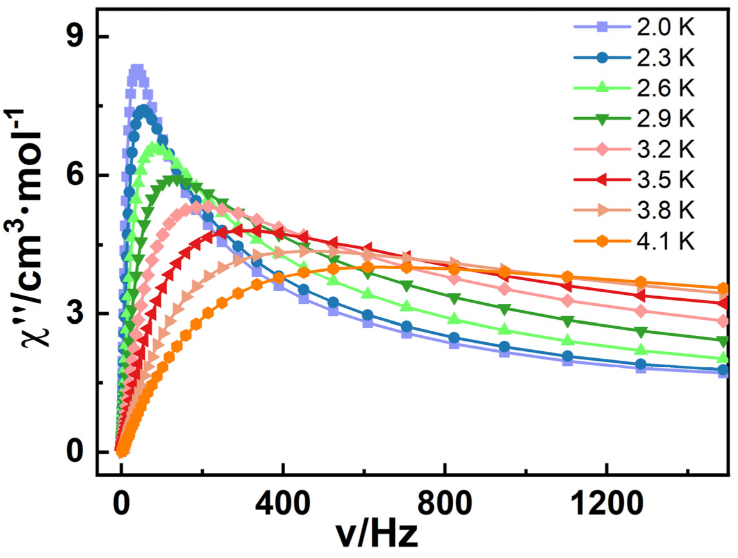

Figure S12. The χ″-ν curves for ***R*-1**.

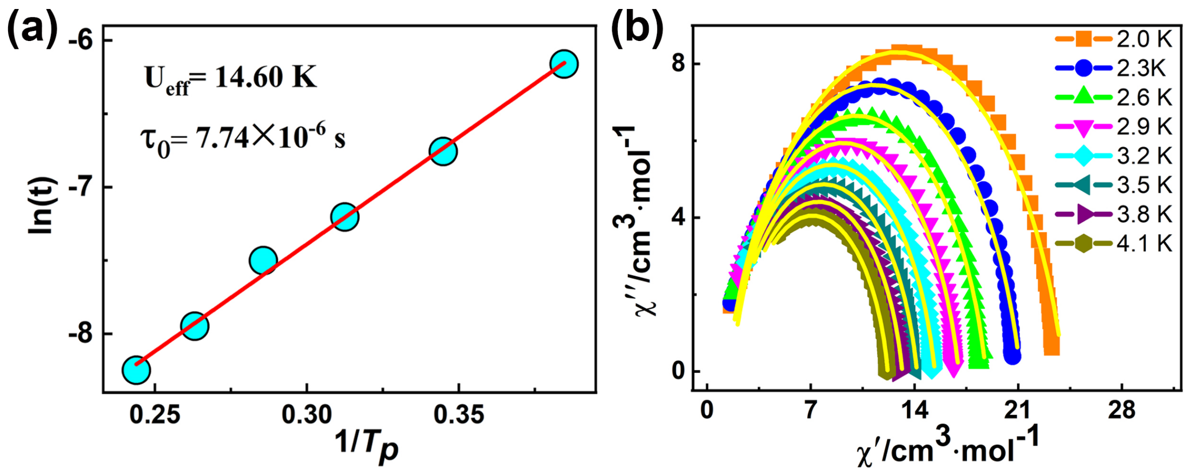

Figure S13. (a) Plot of ln(τ) versus T^{-1} for ***R*-1**, the red solid line is fitted with the Arrhenius law. (b) Cole–Cole plots of ***R*-1** under zero dc field (the yellow solid line represents the least-squares fitting by using CC-FIT software).

Table S5. Linear combination of two modified debye model fitting parameters from 2.0 to 4.1 K at H_{dc} = 0 Oe.

T/K	τ/s	α
2.0	0.00035	0.19018
2.3	0.00253	0.17715
2.6	0.00164	0.17102
2.9	0.00011	0.16663
3.2	0.00070	0.16238
3.5	0.00047	0.15909
3.8	0.00033	0.15236
4.1	0.00024	0.14017

Table S6. The proton conductivity of ***R*-1** at 25 °C under variable relative humidity (RH).

RH / %	σ / S cm^{-1}
60	1.18×10^{-9}
70	6.08×10^{-9}
80	3.15×10^{-8}
90	3.24×10^{-7}
100	7.44×10^{-5}

Table S7. The proton conductivity of ***R*-1** at 100 % RH under variable temperature (°C).

Temperature / °C	σ / S cm^{-1}
25	7.44×10^{-5}
30	8.51×10^{-5}
35	9.44×10^{-5}
40	1.29×10^{-4}
50	1.34×10^{-4}

Table S8. Comparison of the properties of proton conduction, single molecule magnet (SMM) and magneto-optical Faraday effect of *R*-**1** with that of complex based on Schiff ligands. σ represents proton conductivity and RH stands for relative humidity.

Compounds	U_{eff}/k (K)	Conductivity (S cm^{-1})	$\lvert g_{max(MCD)} \rvert$ (T^{-1})	References
(DyCu$_2$[*RR*/*SS* -L]$_2$[H$_2$O]$_3$)·(NO$_3$)$_3$·(H$_2$O) (***R*-1** and ***S*-1**)	17.70 (***R*-1**)	1.34 × 10^{-4} under 50 °C and 98% (***R*-1**)	0.435 (***R*-1**) 0.433 (***S*-1**)	This work
[Cu$_6$Dy$_3$(*R*-L)$_6$(OH)$_6$(H2O)$_6$](ClO$_4$)(NO$_3$)$_2$·4.75H$_2$O·8.5MeOH (***R*-1** and ***S*-1**)	19.5(0.6) (***R*-1**)	4.77 × 10^{-6} under 80 °C and 100% RH (***R*-1**)	0.58 (***R*-1**) 0.58 (***S*-1**)	*Inorg. Chem. Front.*, **2023**, 10.1039/d3qi00634d
